# Knowledge, attitude and practice of Sari birth cohort members during early weeks of COVID-19 outbreak in Iran

**DOI:** 10.1186/s12889-021-11039-6

**Published:** 2021-05-25

**Authors:** Leila Shahbaznejad, Mohammad Reza Navaeifar, Faeze Sadat Movahedi, Fatemeh Hosseinzadeh, Seyed Alireza Fahimzad, Zahra Serati Shirazi, Mohammad Sadegh Rezai

**Affiliations:** 1 Pediatric Infectious Diseases Research Center, Communicable Diseases Institute, Mazandaran University of Medical Sciences, Sari, Iran; 2 Student Research Committee, Mazandaran University of Medical Sciences, Sari, Iran; 3 Pediatric Infections Research Center, Shahid Beheshti University of Medical Sciences, Tehran, Iran; 4 Department of Pediatrics, Division of Pediatric Intensive Care, Faculty of Medicine, Nemazee Hospital, Shiraz University of Medical Sciences, Shiraz, Iran

**Keywords:** COVID-19, Knowledge, Attitude, Practice, Sari birth cohort

## Abstract

**Background:**

It has been determined that the coronavirus disease 2019 (COVID-19) pandemic needs social distancing and proper measures to prevent its spreading. This study aimed to determine COVID-19 knowledge, attitude, and practice among Sari Birth Cohort (SBC) members.

**Methods:**

In this cross-sectional study linked to the SBC in north of Iran, mothers living in Sari and its suburbs from March 28 to April 8, 2020 were evaluated. The measurement tool was an online researcher-made, self-reported knowledge, attitude, and practice questionnaire related to COVID-19.

**Results:**

In total, 1449 mothers with a mean age of 31.51 ± 5.73 years participated. Of them, 82.4% had good knowledge (98.6% in healthcare workers and 79.2% in housewives, *p* = 0.000). Most of them were worried about spread of the disease in the country (97.4%) and agreed that COVID-19 will finally be successfully controlled around the world (72.2%). Sleep disturbance was reported in 42.7% of mothers. Eighty-eight percent of cases wore masks and gloves when leaving home, 99.4% washed their hands frequently while 12.9% went to any crowded places. People with better knowledge followed safer practices (*p* = 0.000) and were more worried about the spread of the disease in the country and infection (among themselves and their first-degree relatives) (*p* = 0.000).

**Conclusions:**

Most of the SBC members had a good level of knowledge about COVID-19 but were worried about a long-term pandemic period. They also had good practices regarding the prevention of the disease.

**Supplementary Information:**

The online version contains supplementary material available at 10.1186/s12889-021-11039-6.

## Background

Last decade witnessed the outbreak of many life-threatening infectious diseases including MERS (Middle East Respiratory Syndrome), Ebola, SARS (Severe Acute Respiratory Syndrome) and more recently, COVID-19 (Coronavirus Infectious Disease-19) which is associated with SARS-CoV2 [1]. COVID-19 infection was first reported in Wuhan, China in December 2019, and is spreading around the world with approximately 125,436,255 confirmed cases and over 2,756,767 deaths as of March 25, 2021 and infects both adults and children with different clinical characteristics [2–4]. Hence, the World Health Organization (WHO) declared it a new pandemic [5].

The reported mortality rate varies in different countries and also in different regions within, but WHO data on the cumulative number of deaths to March 3, 2021 estimated globally a 5.6% mortality rate of COVID-19 infection [6]. Significant efforts have been made to develop therapeutic interventions against the coronavirus infection. The current approach to coronavirus disease management is unclear and sometimes disorganized, but mainly focuses on supportive care [7].

The COVID-19 infection, as an airborne high consequence of infectious disease, has an incubation period of 0 to 14 days [7]. The symptoms range from mild to severe complications including fever, cough, shortness of breath, pneumonia and respiratory, hepatic, gastrointestinal, renal, cutaneous and neurological complications and finally death occurs in severe cases [7–11]. The key principles for COVID-19 prevention are to identify potential cases as soon as possible, prevent transmission of the infection to other people and avoid direct contact with respiratory secretions and isolating the patients [12].

This novel pandemic needs global attention and coordinated response to the rapidly changing messages about public health and immediate actions to minimize the risk of infection and spread of the virus [13]. Since it is known that COVID-19 may be transmitted even from asymptomatic cases, the risk is multiplied [14]. Communicable disease control is a public health priority [15] and epidemiological studies are necessary for monitoring the public response to the emerging crisis, as well as prevention and control of the spread of the disease [16]. Identifying public health problems and related factors may also help decision-makers take appropriate measures to improve individual or community health and make the proper planning and public policies [17]. Since a better knowledge and understanding about this new human challenge may be presently insufficient, many guidelines are released through local governments and/or WHO, to help countries maintain essential health services during the COVID-19 pandemic. Knowledge, as an essential predictor of attitudes and behaviors, cause advancing intervention strategies to promote the public’s precautionary behaviors in the context of the COVID-19 pandemic [18]. It is well-known that confused comprehension and negative attitudes may cause unnecessary worry and anxiety about emerging communicable diseases. Excessive panic would likely aggravate the epidemic [17]. Proper knowledge, attitude, and practices can play an important role in prevention and control of the diseases [19]. Collective engagement of people in preventive behaviors such as practicing personal hygiene and maintaining social distance, prevention of the disease spread is possible and morbidity and mortality rates decrease [18].

To our knowledge, few studies have assessed the general public knowledge on this novel coronavirus and awareness of its prevention and management in the Iranian population. Knowledge, attitudes and practices of mothers has a key role for prevention and control of the diseases, especially communicable ones. Therefore, this study aimed to assess the knowledge, attitudes and practices of Sari Birth Cohort (SBC) mothers in north of Iran, and its related factors, in the early weeks following the outbreak of the global epidemic. We focused on the role of health literacy and other psychosocial health determinants in understanding risks for COVID-19 and performing preventive behaviors.

## Methods

### Setting and participants

The 2017 Sari Birth Cohort (SBC) is an ongoing, multidisciplinary, longitudinal study linked to multicenter Persian birth cohort running in 5 different provinces of Iran (Sari, Isfahan, Yazd, Semnan, and Rafsanjan cities). It takes its subjects from currently pregnant women living in Sari city and rural areas in Mazandaran province in north of Iran. The SBC aims to investigate the impact of socioeconomic status, lifestyle, diet, occupational and environmental exposures before and during pregnancy and also during early life, on some major health concerns in their ongoing child. By the end of the study, totally 3000 mother-child pairs are expected to be included, and the offspring would be followed up for at least 10 years of age. To date, 2800 mothers have been registered in the SBC.

### Study design

In this cross-sectional survey, which was linked to the SBC study, knowledge, attitudes and practices (KAP) of SBC members were evaluated from March 28 to April 8, 2020, a few weeks after the COVID-19 pandemic began in Mazandaran province, in northern Iran and almost at the same time as the New Year holidays in Iran.

### Measures

A self-administered researcher-made online questionnaire in Persian language was offered to the SBC members via a link to their WhatsApp and/or Telegram accounts (Additional files, English questionnaire). Within 1 week, follow up contact was performed, and a reminder message was sent to non-responders.

The questionnaire consisted of two demographic and KAP questions and was developed based on scientific articles and guidelines for clinical and community management of COVID-19 [13, 20, 21] with a total of 41 questions regarding COVID-19: 4 demographic, 3 disease history, 14 knowledge, 7 attitude and 13 practice questions (Accessory files).

In the knowledge questionnaire, the mothers’ source of information about COVID-19 and their knowledge about the disease transmission routes, prevention, symptoms and treatment options were asked on a true/false basis with an additional “I don’t know” option. A correct answer was assigned one point and an incorrect/unknown answer was assigned zero point. The total knowledge score ranged from 0 to 13 scores and was categorized from poor (score 0–3), average (score 4–8) and good (score 9–13) levels.

Attitudes towards COVID-19 were measured by 7 questions concerning the prevalence of the disease throughout the world, the country, first-degree relatives, sleep disturbance and the perceived severity of the disease.

For the assessment of practices, questions had scores between − 2 to + 2 points, with higher points for more important protective behaviors and lower points for more risky actions. Poor practice referred to a total score of 0 to 8 and good practices were described as a total score of 1 to 12.

An expert panel consisting of an epidemiologist, an infectious disease subspecialist, a pediatrician and a biostatistics specialist approved the validity of the questionnaire. In a test-retest, the Cronbach’s alpha coefficient was 0.71 in our sample, indicating acceptable internal consistency [22]. The ethics committee of Mazandaran University of Medical Sciences approved the study protocol (Ethical code: IR.MAZUMS.REC.1399.7354) and consent for publication was obtained from all participants.

### Statistical analysis

Descriptive statistics (mean ± SD, frequency and percentage) were used for all patient characteristics and survey responses. Frequency of correct knowledge, attitudes and practices were described. Chi-square test was used for knowledge scores, attitudes and practices according to demographic characteristics. Data was analyzed by SPSS software, version 16.0 and *p* values less than 0.05 were considered to be statistically significant. Missing data were not included in analysis.

## Results

A total of 1708 questionnaires were sent to the mothers of SBC from March 28 to April 8, 2020, and 1449 members completed them (response rate = 84.8%). The mean age of the responders was 31.51 ± 5.73 years (range: 18–90 years old). Majority of the mothers were aged between 27 to 36 years (867, 62.6%) and 67.4% were housewives (967 cases). None of the mothers were illiterate and 59.1% (850 mothers) held a Bachelors’s degree or higher education (Table 1). Ninety-one percent (1298 mothers) of them lived in the urban areas. The baseline characteristics of the respondents are presented in Table 1.

**Table 1:** General characteristics and KAP of participants regarding COVID-19

	Frequency		Percent	Knowledge				***p***-value	Practice				***p***-value
				Poor 13(0.9%)	Average 241(16.6%)		Good 1189(82.4%)		Poor 132(9.1%)		Good 1317(90.9%)		
**Age (*n =*** **1385)**	**17–26 years**	261	18.8	3 (1.2%)	66 (25.4%)		191 (73.5%)	0.001^¥^	32 (12.3%)		229 (87.7%)		0.055
	**27–36 years**	867	62.6	6 (0.7%)	129 (14.9%)		729 (84.4%)		77 (8.9%)		790 (91.1%)		
	**37–47 years**	257	18.6	3 (1.2%)	33 (12.9%)		220 (85.9%)		16 (6.2%)		241 (93.8%)		
Occupation* (*n =* **1434)**	**Housewife**	967	67.4	11 (1.1%)	189 (19.6%)		762 (79.2%)	0.000^¥^	103 (10.7%)		864 (89.3%)		0.024^¥^
	Non-healthcare worker*	393	27.4	1 (0.3%)	47 (12.0%)		345 (87.8%)		24 (6.1%)		369 (93.9%)		
	**Healthcare worker**	74	5.2	0	1 (1.4%)		73 (98.6%)		5 (6.8%)		69 (93.2%)		
Level of education** (*n =* **1439)**	**Associate degree & lower**	589	40.9	9 (1.5%)	133 (22.7%)		443 (75.7%)	0.000^¥^	75 (12.7%)		514 (87.3%)		0.000^¥^
	**Bachelor’s degree & higher**	850	59.1	4 (0.5%)	106 (12.5%)		739 (87.0%)		57 (6.7%)		793 (93.3%)		
**Living place (*n =*** **1430)**	**Urban**	1298	90.8	12 (0.9%)	203 (15.7%)		1078 (83.4%)	0.042^¥^	121 (9.3%)		1177 (90.7%)		0.508
	**Rural**	132	9.2	1 (0.8%)	32 (24.2%)		99 (75.0%)		10 (7.6%)		122 (92.4%)		
**Knew someone who died from COVID-19 (*n*** **= 1378)**	**Yes**	784	56.9	5 (0.6%)	104 (13.3%)		673 (86.1%)	0.000^¥^	54 (6.9%)		730 (93.1%)		0.006^¥^
	**No**	594	43.1	4 (0.7%)	131 (22.1%)		459 (77.3%)		66 (11.1%)		528 (88.9%)		
**Knew someone who has been cured from COVID-19 (*n*** **= 1448)**	**Yes**	956	66.0	3 (0.3%)	153 (16.0%)		798 (83.6%)	0.003^¥^	75 (7.8%)		881 (92.2%)		0.019^¥^
	**No**	492	34.0	10 (2.0%)	87 (17.8%)		391 (80.1%)		57 (11.6%)		435 (88.4%)		
**COVID-19 in themselves and their first degree relatives (*n*** **= 1449)**	**Yes**	200	13.8	2 (1.2%)	28 (17.3%)		132 (81.5%)	0.869	18 (9.0%)		182 (91.0%)		0.954
	**No**	1249	86.2	11 (0.9%)	213 (16.6%)		1057 (82.5%)		114 (9.1%)		1135 (90.9%)		
**Knowledge**	**Poor**	–	–	–	–		–	–	6 (46.2%)		7 (53.8%)		0.000^¥^
	**Average**	–	–	–	–		–		37 (15.4%)		204 (84.6%)		
	**Good**	–	–	–	–		–		86 (7.2%)		1103 (92.8%)		
	Attitude***												
	**A1**			***p*-value**	**A2**		***p*-value**	**A3**		***p*-value**	**A4**		***p*-value**
	**Yes**		**No**		**Yes**	**No**		**Yes**	**No**		**Yes**	**No**	
**Age (*n =*** **1385)**	**17–26 years**	252 (96.9%)	8 (3.1%)	0.851	169 (86.7%)	26 (13.3%)	0.000^¥^	145 (79.2%)	38 (20.8%)	0.156	48 (35.3%)	88 (64.7%)	
	**27–36 years**	836 (97.5%)	21 (2.5%)		639 (95.1%)	33 (4.9%)		494 (82.3%)	106 (17.7%)		154 (32.4%)	321 (67.6%)	0.816
	**37–47 years**	249 (97.3%)	7 (2.7%)		192 (95.5%)	9 (4.5%)		158 (86.8%)	24 (13.2%)		44 (33.6%)	87 (66.4%)	
Occupation* (*n =* **1434)**	**Housewife**	938 (97.8%)	21 (2.2%)	0.347	678 (92.1%)	58 (7.9%)	0.012^¥^	556 (84.9%)	99 (15.1%)	0.036^¥^	163 (33.6%)	322 (66.4%)	
	**Non-healthcare worker**	375 (96.6%)	13 (3.4%)		298 (97.1%)	9 (2.9%)		222 (78.2%)	62 (21.8%)		70 (30.3%)	161 (69.7%)	0.669
	**Healthcare worker**	71 (95.9%)	3 (4.1%)		59 (92.2%)	5 (7.8%)		47 (79.7%)	12 (20.3%)		17 (31.5%)	37 (68.5%)	
Level of education** (*n =* **1439)**	**Associate degree & lower**	562(96.4%)	21(3.6%)	0.068	407(91.9%)	36(8.1%)	0.070	342(84.7%)	62(15.3%)	0.187	124(43.7%)	160(56.3%)	0.000^¥^
	**Bachelor’s degree & higher**	826(98.05%)	17(2.0%)		632(94.6%)	36(5.4%)		487(81.4%)	111(18.6%)		128(26.2%)	360(73.8%)	
**Living place(*n =*** **1430)**	**Urban**	1251(97.4%)	34(2.6%)	0.795	936(94.0%)	60(6.0%)	0.200	734(82.4%)	157(17.6%)	0.255	220(31.2%)	485(68.8%)	0.001^¥^
	**Rural**	128(97.0%)	4(3.0%)		99(90.8%)	10(9.2%)		92(86.8%)	14(13.2%)		33(51.6%)	31(48.4%)	
**Knew someone who died from COVID-19 (*n*** **= 1378)**	**Yes**	755(97.4%)	20(2.6%)	0.957	570(94.2%)	35(5.8%)	0.430	451(81.4%)	103(18.6%)	0.142	147(32.5%)	306(67.5%)	0.852
	**No**	577(97.5%)	15(2.5%)		427(93.0%)	32(7.0%)		346(85.0%)	61(15.0%)		97(33.1%)	196(66.9%)	
**Knew someone who has been cured from COVID-19 (*n*** **= 1448)**	**Yes**	924(97.8%)	21(2.2%)	0.159	698(93.9%)	45(6.1%)	0.361	561(82.9%)	116(17.1%)	0.973	176(32.5%)	366(67.5%)	0.669
	**No**	471(96.5%)	17(32.5%)		346(92.5%)	28(7.5%)		274(82.8%)	57(17.2%)		81(34.0%)	157(66.0%)	
**COVID-19 in themselves and their first degree relatives (*n*** **= 1449)**	**Yes**	192(97.5%)	5(2.5%)	0.916	138(89.0%)	17(11.0%)	0.016^¥^	106(79.1%)	28(20.9%)	0.218	38(33.0%)	77(67.0%)	0.981
	**No**	1204(97.3%)	33(2.7%)		906(94.2%)	56(5.8%)		729(83.4%)	145(16.6%)		219(32.9%)	446(67.1%)	
**Knowledge**	**Poor**	4(36.4%)	7(63.6%)	0.000^¥^	7(87.5%)	1(12.5%)	0.234	4(80.0%)	1(20.0%)	0.931	1(20.0%)	4(80.0%)	0.823
	**Average**	230(96.6%)	8(3.4%)		161(91.0%)	16(9.0%)		130(83.9%)	25(16.1%)		32(32.7%)	66(67.3%)	
	**Good**	1161(98.1%)	23(1.9%)		876(94.1%)	55(5.9%)		701(82.8%)	146(17.2%)		224(33.1%)	453(66.9%)	
**Practice**	**Poor**	120(95.2%)	6(4.8%)	0.122	98(91.6%)	9(8.4%)	0.409	76(82.6%)	16(17.4%)	0.951	15(27.8%)	39(72.2%)	0.402
	**Good**	1276(97.6%)	32(2.4%)		946(93.7%)	64(6. 3%)		759(82.9%)	157(17.1%)		242(33.3%)	484(66.7%)	
	Attitude***												
	**A5**			***p*-value**	**A6**		***p*-value**	**A7**					***p*-value**
	**Yes**		**No**		**Yes**	**No**		**A**		**B**	**C**	**D**	
**Age(*n =*** **1385)**	**17–26 years old**	251(96.5%)	9(3.5%)	0.567	104(40.0%)	156(60.0%)	0.060	90(35.4%)		105(41.3%)	43(16.9%)	16(6.3%)	0.050
	**27–36 years old**	836(97.3%)	23(2.7%)		357(41.6%)	501(58.4%)		301(35.5%)		385(45.5%)	118(13.9%)	43(5.1%)	
	**37–47 years old**	252(98.1%)	5(1.9%)		126(49.2%)	130(50.8%)		113(44.8%)		103(40.9%)	23(9.1%)	13(5.2%)	
Occupation*(*n =* **1434)**	**House keeper**	934(97.2%)	27(2.8%)	0.710	399(41.6%)	560(58.4%)	0.443	341(36.3%)		402(42.8%)	134(14.3%)	63(6.7%)	0.009^¥^
	**Non-Healthcare worker**	377(96.6%)	12(3.1%)		177(45.4%)	213(54.6%)		159(41.3%)		160(41.6%)	50(13.0%)	16(4.2%)	
	**Healthcare worker**	73(98.6%)	1(1.4%)		32(43.2%)	42(56.8%)		21(28.4%)		46(62.2%)	5(6.8%)	2(2.7%)	
Level of education** (*n =* **1439)**	**Associate degree & lower**	563(96.4%)	21(3.6%)	0.129	266(45.7%)	316(54.3%)	0.061	224(39.6%)		205(36.2%)	88(15.5%)	49(8.7%)	0.000^¥^
	**Bachelor’s degree & higher**	826(97.8%)	19(2.2%)		344(40.7%)	501(59.3%)		301(35.9%)		404(48.2%)	101(12.1%)	32(3.8%)	
**Living place(*n =*** **1430)**	**Urban**	1252(97.2%)	36(2.8%)	0.876	559(43.5%)	727(56.5%)	0.161	469(37.1%)		558(44.1%)	166(13.1%)	71(5.6%)	0.557
	**Rural**	128(97.0%)	4(3.0%)		49(37.1%)	83(62.9%)		52(39.7%)		50(38.2%)	21(16.0%)	8(6.1%)	
**Knew someone who died from COVID-19 (*n*** **= 1378)**	**Yes**	758(97.3%)	21(2.7%)	0.994	342(43.9%)	437(56.1%)	0.384	274(35.8%)		343(44.8%)	107(14.0%)	42 (5.5%)	0.310
	**No**	576(97.3%)	16(2.7%)		246(41.6%)	346(58.4%)		235(40.2%)		236(40.3%)	78(13.3%)	36(6.2%)	
**Knew someone who has been cured from COVID-19 (*n*** **= 1448)**	**Yes**	926(97.5%)	24(2.5%)	0.299	422(44.4%)	528(55.6%)	0.068	335(35.8%)		431(46.0%)	123(13.1%)	47(5.0%)	0.037^¥^
	**No**	470(96.5%)	17(3.5%)		246(41.6%)	294(60.6%)		191(40.2%)		183(38.5%)	67(14.1%)	34(7.2%)	
**COVID-19 in themselves and their first degree relatives (*n*** **= 1449)**	**Yes**	195(98.5%)	3(1.5%)	0.224	104(52.8%)	93(47.2%)	0.002^¥^	75(38.7%)		92(47.4%)	23(11.9%)	4(2.1%)	0.085
	**No**	1202(96.9%)	38(3.1%)		509(41.1%)	730(58.9%)		452(37.1%)		522(42.9%)	167(13.7%)	77(6.3%)	
**Knowledge**	**Poor**	8(72.7%)	3(27.63%)	0.000^¥^	4(44.4%)	5(55.6%)	0.933	2(22.2%)		4(44.4%)	3(33.3%)	0(0.0%)	0.005^¥^
	**Average**	226(94.6%)	13(5.4%)		105(43.8%)	135(56.3%)		75(32.5%)		236(42.0%)	78(14.7%)	36(10.8%)	
	**Good**	1162(97.9%)	25(2.1%)		504(42.5%)	682(57.5%)		235(38.4%)		236(43.8%)	78(13.1%)	36(4.8%)	
**Practice**	**Poor**	120(96.0%)	5(4.0%)	0.419	53(42.7%)	71(57.3%)	0.990	58(49.2%)		39(33.1%)	14(11.9%)	7(5.9%)	0.040^¥^
	**Good**	1277(97.3%)	36(2.7%)		560(42.7%)	752(57.3%)		469(36.2%)		575(44.4%)	176(13.6%)	74(5.7%)	

* Occupation: Non-Healthcare worker: Officer: 120(8.4%), teacher: 110(7.7%), other: 163(11.4%)

** Level of education: Associate degree & lower: Illiterate: 0(0.0%), elementary: 45(3.1%), High school: 388(27.0%), Associate degree: 156(10.8%); **Bachelor’s degree & higher:** Bachelor’s degree: 579(40.2%), Master’s degree: 247(17.0%), PhD, MD or above: 24(1.7%)

*** “I don’t Know” isn’t in the analysis

¥: *P* < 0.05 considered being statistically significant

Attitude questions:

A1. Are you worried about the spread of the disease in the country?

A2. Do you agree that COVID-19 will finally be successfully controlled around the world?

A3. Do you have confidence that Iran can win the battle against the COVID-19 virus?

A4. Do you think Iran will develop drug and vaccines sooner than other countries?

A5. Are you worried about get infection in yourself and your first degree relatives?

A6. Is your sleep disturbed with worrying about COVID-19?

A7. In your opinion, which of the following options usually present with coronavirus disease?

Two hundred mothers (13.8%) reported having COVID-19 infection themselves or in their first-degree relatives and about half of them (784, 54%) knew someone who died from COVID-19 while 956 (66%) knew someone who recovered from it. Results for each of the COVID-19 KAP questions are included in Tables 1 and 2. The reported sources of information were television in 653(45.5%), social media in 515(35.9%), medical websites in 198(13.8%), and friends or family members in 69 cases (4.8%). Knowledge levels were good in 1189(82.4%), average in 241(16.6%) and poor in 13(0.9%) of the mothers (Table 2).

**Table 2:** Knowledge, attitude and practice of Sari birth Cohort members about COVID-19

Questions	Options (Each correct answer (bolded), had 1 point), total score: 13		Frequency	Percent
Knowledge				
K1. Which of the following is the main source of your information about the disease?	1. Newspaper		0	0.0
	2. Television		653	45.5
	3. Medical web sites (WHO, etc.)		198	13.8
	4. Social media such as Telegram, WhatsApp and Instagram		515	35.9
	5. Family and friends, Colleagues, etc		69	4.8
K2. The main clinical symptoms of COVID-19 are fever, fatigue, dry cough, and myalgia	1. **True**		1345	93.7
	2. False		30	2.1
	3. I don’t know		61	4.2
K3. Unlike the common cold, nasal congestion, runny nose, and sneezing are less common in persons infected with the COVID-19 virus	1. **True**		967	67.6
	2. False		106	7.4
	3. I don’t know		358	25.0
K4. There is no effective cure for COVID-2019, but early symptomatic and supportive treatment can help most patients recover from the infection	1. **True**		1151	80.3
	2. False		21	1.5
	3. I don’t know		261	18.2
K5. Not all persons with COVID-19 will develop to severe cases. Only those who are elderly, have chronic illnesses, and are obese are more likely to be severe case	1. **True**		1320	91.9
	2. False		32	2.2
	3. I don’t know		85	5.9
K6. Contact with domestic/wild animals would result in the infection by the COVID-19 virus	1. **True**		674	47.1
	2. False		339	23.7
	3. I don’t know		419	29.3
K7. Persons with COVID-19 can be carrier just when they have fever	1. True		108	7.5
	2. **False**		1024	71.2
	3. I don’t know		306	21.3
K8. The COVID-19 virus spreads via respiratory droplets of infected individuals	1. **True**		1265	88.1
	2. False		62	4.3
	3. I don’t know		109	7.6
K9. Ordinary persons can wear general medical masks to prevent the infection by COVID-19	1. **True**		964	67.2
	2. False,		361	25.2
	3. I don’t know		109	7.6
K10. It is necessary for children and infants to take measures to prevent the infection by the COVID-19 virus	1. **True**		1390	96.9
	2. False,		13	0.9
	3. I don’t know		31	2.2
K11. To prevent the infection by COVID-19, individuals should avoid going to crowded places such as train stations and avoid taking public transportations	1. **True**		1417	98.8
	2. False		6	0.4
	3. I don’t know		11	0.8
K12. Isolation and treatment of COVID-19 infected people are effective ways to reduce the spread of the virus	1. **True**		1407	97.9
	2. False		7	98.4
	3. I don’t know		23	1.6
K13. People who have contact with someone infected with the COVID-19 virus should be immediately quarantine for 14 days	1. **True**		1308	91.3
	2. False		41	2.9
	3. I don’t know		84	5.9
K14. Smokers and addicted people can infect with the coronavirus infection	1. **True**		802	55.9
	2. False		240	16.7
	3. I don’t know		392	27.3
	1. I don’t know		316	22.0
**Attitudes**	**Options**			
A1. Are you worried about the spread of the disease in the country?	1. Agree		1396	97.4
	2. Disagree		38	2.6
A2. Do you agree that COVID-19 will finally be successfully controlled around the world?	1. Agree		1044	72.7
	2. Disagree		73	5.1
	3. I don’t know		319	22.2
A3. Do you have confidence that Iran can win the battle against the COVID-19 virus?	1. Agree		835	58.1
	2. Disagree		173	12.0
	3. I don’t know		429	29.9
A4. Do you think Iran will develop drug and vaccines sooner than other countries?	1. Agree		257	17.9
	2. Disagree		522	36.3
	3. I don’t know		658	45.8
A5. Are you worried about get infection in yourself and your family?	1. Agree		1397	97.1
	2. Disagree		41	2.9
A6. Is your sleep disturbed with worrying about COVID-19?	1. Agree		613	42.7
	2. Disagree		823	57.3
A7. In your opinion, which of the following options usually present with coronavirus disease?	1. Mild or no symptoms		527	37.3
	2. Moderate that requiring self-care and rest		614	43.5
	3. Severe requiring hospitalization		190	13.5
	4. fatal disease		81	5.7
**Practices**	Options: Points in parentheses			
P1. In recent days, have you gone to any crowded places?	1. Yes(−1)		184	12.9
	2. No(+ 1)		1245	87.1
P2. In recent days, have you worn a face mask or gloves when leaving home?	1. Yes(+ 1)		1282	89.8
	2. No(0)		145	10.2
P3. Do you wash your hands with soap or liquid hand washing for 20 s when you enter house?	1. Yes(+ 1)		1421	99.4
	2. No(− 1)		9	0.6
P4. Do you disinfect indoors surfaces and handles?	1. Yes(+ 1)		1367	95.3
	2. No(−1)		67	4.7
4P5. If yes, with what solution?	1. chlorinated Bleaching liquids,		608	44.4
	2. Alcohol based surface disinfectant solution		620	45.3
	3. Ordinary alcohol		69	5.0
	4. Industrial alcohol		22	1.6
	4. handwashing or dishwashing liquids		51	3.7
P6. If yes, how many times a day	1. Once		602	44.3
	2. twice		358	26.3
	3. three times		151	11.1
	4. more than 3 times a day		248	18.2
P7. Have you visited your family members during the New Year holidays?	1. Yes(−1)		54	3.8
	2. No(+ 1)		1380	96.2
P8. Have you traveled to other cities during the New Year holidays?	1. Yes(−1)		28	1.9
	2. No(+ 1)		1408	98.1
P9. Which of the following do you do to prevent contamination?	1. Avoid sick people	1. Yes(+ 1)	964	67.1
		2. No(0)	472	32.9
	2. Covering sneeze and cough	1. Yes(+ 1)	908	63.2
		2. No(−1)	528	36.8
	3. Not using public transportation	1. Yes(+ 1)	1023	71.2
		2. No(0)	413	28.8
	4. Not going to work	1. Yes(0)	542	37.7
		2. No(0)	894	62.3
	5. Not going to the hospital	1. Yes(0)	830	57.8
		2. No(0)	606	42.2
	6. Not sending children to school	1. Yes(0)	611	42.5
		2. No(0)	825	57.5
	7. Using traditional or herbal medication	1. Yes(0)	312	21.7
		2. No(0)	1124	78.3
P10. What’s bothering you mostly these days?	1. Fear of my infection,		217	15.2
	2. Fear of relatives’ infection		575	40.2
	3. Frequent news about spread of the disease		264	18.5
	4. News of the death of other peoples or family members		373	26.1
P11. If you have symptoms of Covid-19, where do you go first to diagnose it?	1. Screening website of the university or the Ministry of Health(+ 2)		466	32.6
	2. Family doctor or GP(+ 1)		451	31.5
	3. Emergency or specialized ward od hospital(+ 1)		332	23.2
	4. Private office of specialist (+ 1)		181	12.7
P12. Do you want to be quarantined at home for two weeks if your doctor or healthcare provider recommend it?	1. Yes (+ 1)		1419	98.8
	2. No (−2)		17	1.2
P13. If you are employed, are you able to work remotely at home?	1. Yes		333	70.9
	2. No		137	29.1

Knowledge of younger mothers was significantly lower; 73.5% of mothers between 17 to 26 years had good knowledge, but 84.4% of mothers above 27 years had good knowledge (*p* = 0.001) (Table 1). Knowledge of mothers also differed according to their occupation: 98.6% of healthcare or health-related workers had good knowledge, while this level of knowledge was seen in 79.2% of homemakers (*p* = 0.000) (Table 1). Mothers who lived in urban areas had better knowledge than rural areas (83.4% vs 75%, *p* = 0.042). Good levels of knowledge were statistically higher among mothers with Bachelor’s degree or above (87% vs 75.7%, *p* = 0.000) (Table 1).

The frequency of good level of knowledge was significantly higher in mothers whose main source of information was social media or medical websites compared to television (86% vs 77.9%, *p* = 0.005). Interestingly, mothers who knew someone who had died (86% vs 77.3%, *p* = 0.000) or recovered from COVID-19 (83.6% vs 80%, *p* = 0.003) had a higher frequency of good level of knowledge compared to others (Table 1).

Regarding attitudes, 1396(97.4%) mothers were worried about the spread of the disease in the country (A1), 1397(97.1%) were worried about becoming infected themselves and/or their first-degree relatives (A5). 1044(72.2%) agreed that COVID-19 will finally be successfully controlled around the world (A2), and sleep disturbance (A6) was reported in 613(42.7%) mothers. Participants’ sleep disturbance also varied according to the family history of infection (Tables 1, 2).

The practice of mothers was good in 1317(90.9%) and poor in 132(9.1%) mothers. Regarding high-risk practices in recent days, only 184(12.9%) went to any crowded places (P1), 54(3.8) had visited their family members during the New Year holiday (P7), and 28(1.9%) had traveled to other cities during the New Year holiday (P8). Concerning protective actions, 1282(88.8%) mothers had worn a mask or gloves when leaving home (P2), 1421(99.4%) mothers washed their hands for 20 s when arriving home (P3), and 1367(95.3%) disinfected indoor surfaces and handles regularly (P4) (Table 2).

When asking “If you have symptoms of COVID-19, where do you go first to diagnose it?”, 466(32.6) responded going to the screening website of the university or the Ministry of Health, and others preferred referring to a physician in a public or private setting (Table 2). From a total of 470 employees, 333(70.9%) were able to work remotely at home, 96(89.7%) were teachers or held academic positions and 32(43.8%) were healthcare workers who could work remotely at home.

Fear of relatives’ infection was positive in 575(39.7%), and fear of my infection in 217(15%) mothers (Table 2). Table 2 summarized the practices participants performed for their protection. People who had better knowledge were more worried about the spread of the disease in the country (97.8% of average and good knowledge scores were worried, compared to 36.4% in the weak knowledge group, *p* = 0.000). Further, mothers with average or good levels of knowledge were more worried about the infection of themselves and their first-degree relatives (97% vs 72.7%; *p* = 0.000) (Table 1). People who had better knowledge, practiced better (*p* = 0.000), while 53.8% of poor knowledge people, practiced good (84.6%) and 92.8% of mothers with moderate or good knowledge levels followed safer practices. No statistically significant association was found between attitudes and practices among the participants (*p* > 0.05) (Table 1).

## Discussion

This study was performed 6 weeks after the COVID-19 outbreak and critically affected the area in northern Iran. The current study reports the knowledge, attitudes, and practices (KAP) regarding the pandemic among the SBC members. COVID-19 has created a global pandemic. It is important to encourage the public to adopt precautionary behaviors, which are based on a correct understanding of the epidemic and the appropriate responses necessary among people [17]. Many studies have evaluated the various levels of KAP about the COVID-19 outbreak showing good levels of knowledge among participants [13, 16, 20, 21]. Social media and global networks have been used in various health applications and improve people’s knowledge during pandemics [23].

In this study, most of the mothers had acceptable level of knowledge which may be affected by their age, occupation, location, educational status, and their main source of information. Respondent’s age, occupation, and education level were related to the knowledge level of COVID-19. Contrary to us, in Lee et al.’s study, age was not related to the knowledge [18]. Li et al. [24] reported lower levels of knowledge and practices related to COVID-19 in older respondents while better-educated respondents had higher levels of knowledge and practices. Zhong et al. [21] suggested that knowledge regarding COVID-19 was significantly lower in males, younger ages (16–29 y/o vs older), never married, bachelor’s degree and below and unemployed people. In an Egyptian survey [20], no difference was found between knowledge of males and females, but those who were 50 years and younger, residents of urban areas, and university-educated participants had better knowledge scores. Since all of our participants were married and female, we were unable to compare marital status and gender regarding knowledge, but it can explain the good knowledge score in our study. Also, we found that the knowledge of mothers who were older than 27 years, had higher education levels, and employed mothers were better; perhaps because they use social media or search medical websites seeking the best available information about the disease. As was predictable, the information of healthcare worker mothers was higher than others. Also, Moro et al. found that knowledge of healthcare workers was better than other staff members of hospitals [25]. Clements et al. found that knowledge about COVID-19 was higher in older citizens than younger ones and older people were less likely to attend large gatherings but wearing masks in public was higher in younger people [26]. Better-educated individuals have higher scores because they can process information more quickly, and may be more capable of distinguishing correct information and acting upon it [24].

We found that at the first weeks of COVID-19 outbreak in Iran, 88% of SBC members wore masks and gloves when leaving home. In Clements et al.’s sample in the United States, approximately 76% of people did not wear masks outside the home indicating that a large section of the US public chose to ignore recommendations and it could be the reason for higher prevalence of mortality and morbidity in the world [26]. In China, only 2% of people reported not wearing masks outside the home [21]. Use of masks is an evolving and cultural phenomenon [26].

The present study showed that knowledge of mothers regarding some questions was better; they knew the main clinical symptoms of COVID-19, they agreed that taking measures to prevent the infection is necessary for children and infants, infections may be more severe in some comorbidities, avoided going to crowded places, agreed that isolation and treatment of COVID-19 infected people or quarantine of those who had contact with sick people are important to prevent spreading the virus. Other studies also showed similar good information among people [20, 21, 25]. The knowledge of Chinese citizens was high [21] because of their experiences with the severe acute respiratory syndrome outbreak in the early 2000s and their samples were relatively affluent and highly educated.

We found a high prevalence of misunderstanding regarding the source of infection through contact with wild animals and infection by smokers and addicted people, as only 47.1% of respondents correctly answered the question. Also, the idea that COVID-19 is just transmitted in the febrile period of infection and the necessity of wearing masks to prevent infection were other blind spots of our participants. In Lee et al.’s study [18] which was performed after us, only 27.9% of the participants answered correctly. The contexts behind this misinformation might be due to an inconsistency about wearing masks and transmission of the disease by animals in the literature or social media [21, 25]. Thus, future researches about COVID-19 dispersed across communication platforms to provide accurate and evidence-based information about the disease and prevention measures are suggested.

In this study, mothers older than 27 years, who were not healthcare workers, and those without any family history of infection were more hopeful about the eventual control of the disease in the world. Housewives were more hopeful about control of the disease in the country. In Wuhan, China, 90.8% of respondents agreed that this epidemic will be finally controlled, and this attitude was significantly different regarding the educational level and knowledge about COVID-19 [21].

A considerable number of mothers in this study experienced sleep disturbance and mothers with a family history of infection suffered from sleep problems more frequently. This may be due to concerns about the health of their family members. Rajkumar et al. reported that anxiety, depression and self-reported stress are common psychological problems during the COVID-19 pandemic, and may be associated with disturbed sleep [27].

The present study showed that the practices of mothers were good in 91%. They tried to pay attention to preventive measures heterogeneously, and only 13% of them went to crowded places. Most of them have stayed at home, kept social distancing, and avoided traveling, even during the New Year holiday. Although the government never locked down any city or province completely, all mothers practiced such preventive measures by themselves. In Clements et al.’s study [26], nearly 30% of people reported attending gatherings or going to places with more than 50 people. In Zhong et al.’s study [21], during the lockdown of Hubei province, nearly 3.6% reported going to crowded places and 2% did not wear a mask when leaving home. In their study, adhering to different preventive measures of COVID-19 infection was worst in males and people with lower knowledge scores, single persons, and people who were not residents of Hubei were less possible to wear a mask when leaving home [21].

In this study, knowledge was significantly associated with attitude and practice. Similar to us, in Lee et al.’s study, knowledge directly affected both attitudes and practices [18]. In accordance to us, other studies reported similar associations when performing KAP surveys toward COVID-19 [28, 29].

Our study had some limitations. First, it was a local survey in a city and suburbs. Therefore, the result cannot be generalized to the entire country. Knowledge about COVID-19 is rapidly changing, and what was considered “correct” at the time of this writing may not be “correct” anymore [26]. Since members of SBC were in specified fertility age groups and had access to social media, their knowledge might be higher than other people. We could not evaluate all aspects of KAP and only limited and some important aspects were studied. Evaluation of the psychosocial effects of a pandemic on people and relationships with such important factors on the KAP is recommended.

## Conclusion

Knowledge can play a crucial role in enhancing the practice of public preventive behaviors. This survey is one of the first attempts to study determinants of knowledge, attitude and behaviors in response to the COVID-19 pandemic in northern Iran. Although most of the mothers had good level of knowledge about COVID-19 in this study, there are differences in knowledge based on age, education, living place, and so on. Mothers who knew someone who had died or recovered from COVID-19 had higher Knowledge. They also demonstrated good practices regarding the prevention of the disease. Although practice of the SBC members was good in most cases, most of them were worried about a long-term pandemic period and had some knowledge misconceptions. These results suggest that health authorities need to ensure correct information on COVID-19 prevention and strengthen health interventions, particularly for older and less-educated people, to combat rumors and misinformation and reduce public panic.

## Supplementary Information

**Additional file 1: English questionnaire**. Questionnaire of “Knowledge, attitude and practice of Sari Birth Cohort members during early weeks of COVID-19 outbreak in Iran” research project

## Data Availability

The datasets generated and/or analysed during the current study are not publicly available but are some parts of it is available from the corresponding author on reasonable request.

## Abbreviations

SBC: Sari birth cohort
KAP: Knowledge, attitudes, and practices
COVID-19: Coronavirus infectious disease-19
