# Supplementary material for: Knowledge, attitude and practice of Sari birth cohort members during early weeks of COVID-19 outbreak in Iran
Source: BMC Public Health. 2021 May 25;21:982. doi: 10.1186/s12889-021-11039-6 (PMC8148405; doi:10.1186/s12889-021-11039-6)
Supplement: Supplementary file 1 — Additional file 1: English questionnaire. Questionnaire of “Knowledge, attitude and practice of Sari Birth Cohort members during early weeks of COVID-19 outbreak in Iran” research project [file 12889_2021_11039_MOESM1_ESM.docx]

**Questionnaire of “Knowledge, attitude and practice of Sari Birth Cohort members during early weeks of COVID-19 outbreak in Iran” research project**

1. Age: ……years
2. Occupation: housewife□, Officer□, Healthcare worker□, Teacher□, Other………………
3. Level of education : Illiterate□, elementary□ , High school□, Associate degree□, Bachelor’s degree□, Master’s degree□, PhD□, MD or above□
4. Living place: Rural□ Urban□
5. Have you or any members of your family (father, mother, husband, brother, sister, children) been infected with COVID-19? Yes□ No□
6. Do you know someone who died of the coronavirus infection? Yes□ No□
7. Do you know anyone who has been infected with coronavirus and cured? Yes□ No□

| Questions | Options |
| --- | --- |
| **Knowledge** (each correct answer, (bolded), had 1 point), total score: 0-13 |  |
| K1. Which of the following is the main source of your information about the disease? | Newspaper□ Television□ medical web sites (WHO, etc.) □ Social media such as Telegram, WhatsApp and Instagram□ family and friends, Colleagues, etc□ |
| K2. The main clinical symptoms of COVID-19 are fever, fatigue, dry cough, and myalgia. | **True**, False, I don’t know |
| K3. Unlike the common cold, nasal congestion, runny nose, and sneezing are less common in persons infected with the COVID-19 virus. | **True**, False, I don’t know |
| K4. There is no effective cure for COVID-19, but early symptomatic and supportive treatment can help most patients recover from the infection | **True**, False, I don’t know |
| K5. Not all persons with COVID-19 will develop a severe case of COVID-19. Only those who are elderly, have chronic illnesses, and are obese are more likely to be affected severely. | **True**, False, I don’t know |
| K6. Contact with domestic/wild animals would result in infection by the COVID-19 virus. | **True**, False, I don’t know |
| K7. Persons with COVID-19 can be carriers when they just have a fever. | True, **False**, I don’t know |
| K8. The COVID-19 virus spreads via respiratory droplets from infected individuals. | **True**, False, I don’t know |
| K9. Ordinary persons can wear general medical masks to prevent infection by COVID-19 | **True**, False, I don’t know |
| K10. It is necessary for children and infants to take measures to prevent infection by the COVID-19 virus? | **True**, False, I don’t know |
| K11. To prevent infection by COVID-19, individuals should avoid going to crowded places such as train stations and avoid taking public transportation. | **True**, False, I don’t know |
| K12. Isolation and treatment of COVID-19 infected people are effective ways to reduce the spread of the virus. | **True**, False, I don’t know |
| K13. People who have contact with someone infected with the COVID-19 virus should be immediately quarantined for 14 days. | **True**, False, I don’t know |
| K14. Smokers and addicted people can be infected with COVID-19. | **True**, False, I don’t know |
| **Attitudes** |  |
| A1. Are you worried about the spread of the disease in the country? | Agree, disagree, I don’t know |
| A2. Do you agree that COVID-19 will finally be successfully controlled around the world? | Agree, disagree, I don’t know |
| A3. Do you have confidence that Iran can win the battle against the COVID-19 virus? | Agree, disagree, I don’t know |
| A4. Do you think Iran will develop drugs and vaccines sooner than other countries? | Agree, disagree, I don’t know |
| A5. Are you worried about getting infected yourself or your family members? | Agree, disagree, I don’t know |
| A6. Is your sleep disturbed by worrying about COVID-19? | Agree, disagree, I don’t know |
| A7. In your opinion, which of the following symptoms usually present with COVID-19? | Mild or no symptoms□, Moderate that requiring self-care and rest□, Severe requiring hospitalization□, fatal disease□ |
| **Practices (each point in parentheses, total score from -8 to 12)** |  |
| P1. In recent days, have you gone to any crowded places? | Yes(-1), no(+1) |
| P2. In recent days, have you worn a face mask or gloves when leaving home? | Yes(+1), no(0) |
| P3. Do you wash your hands with soap or liquid hand washing detergent for 20 seconds when you enter house? | Yes(+1), no(-1) |
| P4. Do you disinfect indoors surfaces and handles? | Yes(+1), no(-1) |
| P5. If yes, with what solution? | chlorinated  Bleaching liquids□, Alcohol based surface disinfectant solution□, Ordinary alcohol□, Industrial alcohol□, hand washing or dishwashing liquids□ |
| P6. If yes, how many times a day | Once□, twice□, three times□, more than 3 times a day□ |
| P7. Have you visited your family members during the New Year holidays? | Yes(-1), no(+1) |
| P8. Have you traveled to other cities during the New Year holidays? | Yes(-1), no(+1) |
| P9. Which of the following do you do to prevent contamination? (more than one selection is possible)   - Avoid sick people, selected: +1, no answer: 0 - Cover sneezing and coughing, selected: +1, no answer: -1 - Not using public transportation, selected: +1, no answer: 0 - Not going to work, any answer: 0 - Not going to the hospital, any answer: 0 - Not sending children to school, any answer: 0 - Use of traditional or herbal medication, any answer: 0 | Selection from a list |
| P10.What's bothering you most about COVID-19 these days? | Fear of my infection□, Fear of relatives’ infection□, Frequent news about spread of the disease□, News of the death of other peoples or family members□ |
| P11. If you have symptoms of Covid-19, where do you go first to diagnose it? | Screening website of the university or the Ministry of Health(+2)□, Family doctor or GP(+1)□, Emergency or specialized ward od hospital(+1)□, Private office of specialist(+1)□ |
| P12. Would you agree to be quarantined at home for two weeks (14 days) if your doctor or health care provider recommended it? | Yes(+1), No(-2) |
| P13. If you are employed, are you able to work remotely at home? | Yes□ No□ |
